# Bioanalysis of aminoglycosides using high-performance liquid chromatography

**DOI:** 10.5599/admet.1183

**Published:** 2022-02-14

**Authors:** Seth K. Amponsah, Joseph A. Boadu, Daniel K. Dwamena, Kwabena F. M. Opuni

**Affiliations:** 1 Department of Medical Pharmacology, University of Ghana Medical School, University of Ghana, Ghana; 2 Department of Pharmaceutical Chemistry, School of Pharmacy, University of Ghana, Ghana

**Keywords:** PK studies, Therapeutic drug monitoring, Biological matrices

## Abstract

Aminoglycosides are broad-spectrum antibiotics used in the treatment of gram-negative bacterial infections. Due to their nephrotoxic and ototoxic potential (narrow therapeutic index), the use of aminoglycoside for clinical indications requires monitoring. The objective of this review was to identify relevant literature reporting liquid chromatographic methods for the bioanalysis of aminoglycosides in both preclinical and clinical settings/experiments. Data on liquid chromatographic methods were collected from articles in an online academic database (PubMed, Science Direct, Scopus, and Google Scholar). All 71 articles published from 1977 to 2020 were included in the review. Reversed-phase liquid chromatography was the most used method for the bioanalysis of aminoglycosides. Fluorescence or ultraviolet detection methods were mostly used from 1977 to 2002 (51 articles), while mass spectrometry was predominantly used as a detector from 2003 to 2020 (15 articles). Sixty-seven articles reported calibration ranges, which varied significantly for the various drugs assayed: some in the range of 0.1-0.5 ng/mL and others 1250-200000 ng/mL. Also, 61 articles reported R^2^ values (0.964-1.0) for almost all analytes under consideration. Sixty-three articles reported percent recoveries mostly between 61.0 % to 114.0 %, with only two articles reporting recoveries of 4.9 % and 36 %. Out of the 71 reviewed articles, 56 reported intermediate precision values ranging between 0.331 % to 19.76 %, which is within the acceptable limit of 20 %. This review will serve as a guide for research and/or routine clinical monitoring of aminoglycosides in biological matrices.

## Introduction

Aminoglycosides are broad-spectrum antibiotics that are used in the treatment of gram-negative bacterial infections [1,2]. Aminoglycosides elicit their pharmacological effect by binding to the 16S rRNA ribosomal subunit of bacteria and blocking mRNA translation, altering protein synthesis. Structurally, this class of antibiotics has amino sugars in their core connected via glycosidic linkages to a dibasic aminocyclitol [3]. Aminoglycosides are relatively hydrophilic, hence, rarely undergo biotransformation *in vivo*. Aminoglycosides, to some extent, bind to plasma proteins and are excreted entirely unchanged in urine [4]. Streptomycin, netilmicin, tobramycin, kanamycin, spectinomycin, gentamicin, neomycin, amikacin, and paromomycin are examples of aminoglycosides [5,6].

Aminoglycosides are known to possess nephrotoxic and ototoxic potentials, limiting their clinical use [2]. In addition, due to their narrow therapeutic index, therapeutic monitoring is required for aminoglycosides. Especially among patients with underlying renal problems, monitoring aminoglycosides in biological matrices ensures optimal therapy and reduces toxicity [7].

There are several validated bioanalytical methods used to quantitatively determine levels of aminoglycosides in biological matrices. Microbiological assays, radioimmunoassay (RIA), radioenzymatic assays, fluorescence polarization immunoassay (FPIA), high-performance liquid chromatography (HPLC), gas chromatography (GC) and mass spectrometric techniques are some common examples [8-12]. Of these methods, HPLC is the most preferred or routinely used [13]. Indeed, several studies have used HPLC for the bioanalysis of aminoglycosides [14-16]. Although there are recently published reviews on aminoglycosides, the focus of these reviews have been a) pre-treatment and analysis methods of aminoglycosides in food [17], b) challenges in the development of analytical test procedures for aminoglycosides [18], and c) determination of kanamycin by high-performance liquid chromatography [19].

**Figure 1.**
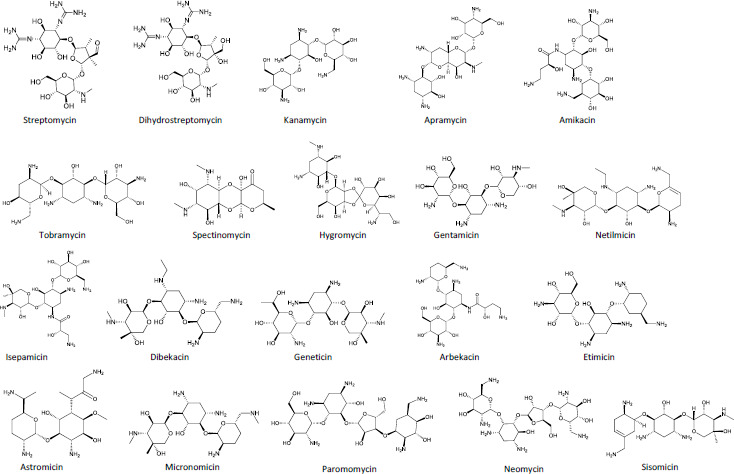
Structures of the 20 aminoglycosides reported by the various articles reviewed

The current review focuses on liquid chromatographic methods employed for the bioanalysis of aminoglycosides. A total of 20 aminoglycosides were reported by 71 articles (total number reviewed). The aminoglycosides were gentamicin [20], netilmicin [21], amikacin [22], tobramycin [23], dibekacin [24], sisomicin [25], astromicin [25], micronomicin [25], kanamycin [26], streptomycin [27], neomycin [14], isepamicin [28], geneticin [29], dihydrostreptomycin [30], paromomycin [31], apramycin [5], hygromycin [5], etimicin [32], arbekacin [33], and spectinomycin [34]. The aforementioned aminoglycosides obtained from either natural products or semi-synthetic derivatives of soil actinomycetes notably *Streptomyces* have suffix -mycin (examples are streptomycin, dihydrostreptomycin, kanamycin, apramycin, paromomycin, neomycin, tobramycin, spectinomycin, and hygromycin); and those obtained from other actinomycetes notably *Micromonospora* have the suffix -micin (examples are gentamicin, netilmicin, isepamicin, sisomicin, etimicin, geneticin, astromicin, and micronomicin). There are other exceptions, such as amikacin, arbekacin, and dibekacin. The constitute structures of these 20 aminoglycosides are presented in Figure 1. In the review, gentamicin was the most reported aminoglycoside (24 articles). This is not surprising since gentamicin is often used clinically because of its low cost and high efficacy against gram-negative aerobes. Fifty (50) articles reported bioanalysis of at least one of the 20 aminoglycosides, whilst 21 articles reported analysis of more than one aminoglycoside. The highest number of aminoglycosides simultaneously assayed was 13 [5].

Although the list of articles used in this review may not be exhaustive, suitable liquid chromatographic conditions used in assaying aminoglycosides in biological matrices have been identified. Performance metrics of the various liquid chromatographic assays have also been appraised. Also, highlights of current procedures, scope, characteristics, and limitations of chromatographic methods used in assaying aminoglycosides in biological matrices have been provided. Although this is not a systematic review, it will serve as a comprehensive reference for subsequent related research that may involve the assay of aminoglycosides.

## Methods

This study reviewed relevant and accessible articles on liquid chromatographic assays of aminoglycosides in biological matrices from 1977 to 2020. Articles were retrieved from journals in online academic databases (PubMed, Science Direct, Scopus, and Google Scholar) and limited to only the English language. Keywords used during the search were aminoglycosides, assay, HPLC, plasma, serum, milk, cerebrospinal fluid, and urine. The searched terms used were ‘‘chromatographic assay’’, ‘‘aminoglycosides’’ and ‘‘biological matrix’’. Articles were excluded if they were not pertinent.

## Aminoglycosides

Sixty-seven (67) out of the 71 articles reported the use of HPLC in the bioanalysis of aminoglycosides, while four articles used ultra-performance liquid chromatography (UPLC) [35]. The relevant aspect of liquid chromatographic conditions used in the various articles, such as matrix, sample preparation, flow rate, column selection, mobile phase, and detection, have been summarized (Table 1).

## Sample cleanup

The matrix in which a drug is found can affect bioanalysis. Also, matrices can compromise the sensitivity and selectivity of bioanalysis methods [86-89], which is deemed a “matrix effect”. The matrix effect could be due to endogenous or exogenous agents. Some endogenous substances include salts, carbohydrates, amines, urea, lipids, peptides, and metabolites [90,91]. Exogenous substances may include anticoagulants, blood preservatives, and mobile-phase additives such as buffer salts [92,93]. Sample cleanup is required to isolate analyte(s) of interest from a matrix.

Sample cleanup tends to reduce or remove matrix components and concentrate the analyte(s). This process improves assay sensitivity and selectivity. An optimal sample cleanup system should be capable of minimizing matrix effect while ensuring reliable extraction recovery [94]. Basically, there are three sample preparation methods that can be applied during HPLC bioanalysis of aminoglycosides; protein precipitation (PPT), solid-phase extraction (SPE), and liquid-liquid extraction (LLE) [4]. All the articles reviewed provided sample preparation methods; 41 used PPT, 33 SPE, and 4 LLE. One article reported the use of ultrafiltration as a sample preparation method [56], 6 articles reported a combination of PPT and SPE [25,42,46,47,61,62], and 1 article used a combination of PPT, SPE, and LLE [72].

Solvents that were used for PPT included acetonitrile for the extraction of netilmicin, amikacin, tobramycin, gentamicin, etimicin, neomycin, and isepamicin [6,15,21,23,32,37-40,42,68,73-76,78,79,82,86]; trichloroacetic acid for the extraction of geneticin, spectinomycin, tobramycin, gentamicin, kanamycin, hygromycin, apramycin, streptomycin, dihydrostreptomycin, amikacin, neomycin, and paromomycin [29,34,35,57,61,72,81]; methanol for the extraction of geneticin, amikacin, tobramycin, sisomicin, and netilmicin [29,41,48]; perchloric acid for the extraction of kanamycin, dibekacin, arbekacin, and streptomycin [26,33,49,51]; formic acid for the extraction of amikacin [84,85]; and methylene chloride for the extraction of isepamicin [62]. PPT aids in reducing interference during derivatization [7], and this may have accounted for its use by 41 articles.

SPE is useful in isolating polar analytes such as aminoglycosides. SPE has also been proven to be useful when the volume of a matrix is high [4]. Preparation of matrices containing aminoglycosides using SPE tends to give well reproducible recovery [95]. Despite these merits, SPE is relatively expensive compared to PPT. In this review, 33 of the articles reported the use of SPE as a sample preparation technique for the extraction of gentamicin, amikacin, tobramycin, sisomicin, netilmicin, astromicin, micronomicin, streptomycin, neomycin, isepamicin, dihydrostreptomycin, kanamycin, paromomycin, spectinomycin, apramycin, and hygromycin in biological matrices.

Also, four articles adopted the LLE as a sample preparation technique for the extraction of netilmicin, gentamicin, amikacin, and dihydrostreptomycin. Aminoglycosides are hydrophilic in nature, and LLE may not be ideal for extraction.

## Chromatographic conditions

All articles reviewed reported chromatographic conditions, and these are highlighted in this review.

### Mode of chromatography

The most-reported mode of chromatography used for bioanalysis of aminoglycosides was reversed-phase HPLC (66 articles). This may be due to the polarity of the analytes of interest [96]. Other modes of chromatography for the separation of aminoglycosides used were hydrophilic interaction liquid chromatography (2 articles) for the analysis of amikacin, gentamicin, kanamycin, neomycin, paromomycin, and tobramycin [31,69]; hydrophobic interaction chromatography (1 article) for the analysis of neomycin [14]; and ion-exchange chromatography (1 article) for the analysis of gentamicin [40]. One article reported the use of mixed-mode chromatography using both reversed-phase and normal chromatography for the analysis of amikacin [41].

### Stationary phase

The selection of a chromatographic mode of separation is dependent on the choice of the column. In this review, 63 articles reported the use of C_18_ columns for the separation of any of the twenty aminoglycosides; while 3 other articles used C_8_ columns for the separation of streptomycin, dihydrostreptomycin, and amikacin [30,47,65]. Also, ion exchange for the separation of gentamicin [40]; cyano for the separation of tobramycin [37]; shielded hydrophobic phase for the separation of neomycin [14]; silica for the separation of amikacin [41]; and hydrophilic interaction columns for the separation of amikacin, gentamicin, kanamycin, neomycin, paromomycin, and tobramycin [31,69] have been reported. This implies that C_18_ columns may be the most appropriate for liquid chromatographic bioanalysis of aminoglycosides. In this review, columns were kept at temperatures ranging from 22 °C to as high as 100 °C [41]. Overall, the most widely used column temperature was 25 °C for the separation of netilmicin, amikacin, tobramycin, gentamicin, dibekacin, sisomicin, isepamicin, geneticin, streptomycin, dihydrostreptomycin, and neomycin [6,24,28-30,42,61,64,68,73]; followed by 30 °C for the separation of neomycin, streptomycin, dihydrostreptomycin, amikacin, kanamycin, paromomycin, tobramycin, spectinomycin, apramycin, hygromycin, gentamicin, netilmicin, etimicin, and isepamicin [5,16,32,72,78,81,82]; and 50 °C for the separation of sisomicin, netilmicin, astromicin, micronomicin, streptomycin, gentamicin, tobramycin, and amikacin [25,27,43,46,47,50].

### Internal standard

Internal standards are normally employed to offset injection volume errors and/or losses during sample extraction [97]. Forty-four (44) articles used internal standards, which include N-acetyl gentamicin C1 [22], tobramycin [22,24,25,44,55,83], amikacin [22,76,78], gentamicin C2 [37], netilmicin [25,39,42,48,58,66], kanamycin [45,47,73,84,85], sisomicin [25,46,48,68,75], neamine [24], gentamicin C1a [6,24], astromicin [25], dihydrostreptomycin [27], dibekacin [28,33,62], gentamicin [59], neomycin [16,77], naphthalene [65], anthracene [67], streptomycin [72], quinoxaline [79], kanamycin B [81], and deuterated paromomycin acetic acid [6]. Of the internal standards used, 13 were aminoglycosides, 3 were modified aminoglycosides [6,22,81], and 4 were not aminoglycosides or congeners of aminoglycosides [24,65,67,79].

### Mode of elution

Although the mobile phase is not directly responsible for chromatographic separation, it can affect chromatographic resolution, selectivity, and efficiency [96]. The selection of a suitable mobile phase for the HPLC system is dependent on the physicochemical properties of the analyte [98]. Since reversed-phase chromatography was reported by most articles, mobile phase solvents used consisted of an aqueous buffer and a non-ultraviolet active water-miscible organic solvent [96]. The solvents included acetonitrile for the separation of netilmicin, gentamicin, and amikacin [21,39,40,47,60]; methanol and water mixture for the separation of gentamicin, amikacin, tobramycin, netilmicin, dibekacin, and sisomicin [20,22,24,36,44]; and heptanesulphonic acid for separation of gentamicin, and netilmicin [58,59]. Other solvents such as tetrahydrofuran for the separation of amikacin [15]; octanesulphonate for the separation of tobramycin, sisomicin, and netilmicin [48]; and ethylene glycol for the separation of sisomicin [54] were also reported. In the case where ionisable analytes were present, the pH of the mobile phase had a significant effect on the ionization state(s) of the analyte(s), which eventually affected resolution. Thus, the buffer reported by most articles were aqueous, which included tris buffer [23], sodium acetate [24], and potassium phosphate buffer [15]. Basic analytes such as aminoglycosides are protonated at low pH when ionized.

Poor elution of analytes could contribute to peak broadening [96,99]. Out of 71 articles reviewed, 50 employed isocratic elution for the separation of streptomycin, gentamicin, amikacin, dihydrostreptomycin, kanamycin, netilmicin, isepamicin, sisomicin, paromomycin, neomycin, tobramycin, etimicin, and dibekacin; whilst the remaining 21 used gradient elution for the separation of streptomycin, gentamicin, amikacin, dihydrostreptomycin, kanamycin, netilmicin, apramycin, isepamicin, sisomicin, paromomycin, geneticin, neomycin, tobramycin, arbekacin, spectinomycin, hygromycin, dibekacin, astromicin, and micronomicin. For complex multicomponent samples, gradient elution is used since all components cannot be eluted between the retention factor of 1 and 10. An isocratic mode is sufficient if the measured ratio is less than 0.25, however, if the ratio is greater than 0.25, then an elution gradient is deemed suitable [96,99].

### Derivatization and mode of detection

Of the 71 articles reviewed, fluorescence (36 articles), UV (15 articles), mass spectrometry (15 articles), resonance Rayleigh scattering (1 article) [32], pulsed electrochemical (1 article) [15], chemiluminescence (1 article) [71], and evaporative light scattering (1 article) [70] were used in the detection of aminoglycosides.

Fluorescence (~50 %) was the most used method for the detection of streptomycin, gentamicin, amikacin, dihydrostreptomycin, kanamycin, netilmicin, isepamicin, sisomicin, neomycin, tobramycin, dibekacin, astromicin, and micronomicin. However, non-fluorescence drugs such as aminoglycosides are mostly difficult to detect by this mode due to the absence of a fluorophore. This shortcoming can be mitigated by derivatization [100]. Additionally, the mobile phase used must be selected with care, as highly polar solvents or halide ions can quench fluorescence. It is noteworthy that fluorescence is mostly preferred to UV detection due to its high sensitivity and selectivity [101]. Aminoglycosides assayed in various studies with fluorescence detectors were achieved at an excitation wavelength range of 220 nm [21] to 490 nm [77] and emission wavelength range of 415 nm [57] to 531 nm [74].

UV-visible detectors are not easily influenced by the mobile phase and surrounding temperature [102]. UV-visible detectors interact with compounds containing chromophores. Since aminoglycosides do not have chromophores, derivatization of these compounds is necessary for their detection. UV-visible was used for the detection of gentamicin, amikacin, tobramycin, streptomycin, netilmicin, and geneticin [6,27,29,38,39,41,45,47,58,64,65,67]. In this review, the UV wavelength range used for the detection of aminoglycosides was 195 nm [27] to 365 nm [6,38,45,64].

In all the 51 articles that used fluorescence or UV detection, only 3 articles did not use derivatization for the detection of streptomycin, dihydrostreptomycin, and sisomicin [27,30,54]. The most common derivatizing agent used in the bioanalysis of aminoglycosides was o-phthalaldehyde; reported by 24 articles for the derivatization of gentamicin, amikacin, kanamycin, netilmicin, isepamicin, sisomicin, neomycin, tobramycin, dibekacin, astromicin, and micronomicin [6,14,20,22-26,28,36,37,43,44,48,50,52,53,55,57-59,61,62,66]. Dansyl chloride for the derivatization of netilmicin [21]; 1-fluoro-2,4-dinitrobenzene for the derivatization of geneticin, gentamicin, and amikacin [29,38,41,45,56,64]; 7-fluoro-4-nitrobenz-2-oxa-1,3-diazole for the derivatization of amikacin [74]; benzene sulphonyl chloride for the derivatization of gentamicin [39]; 6-aminoquinolyl-N-hydroxysucciminidylcarbamate for the derivatization of isepamicin [76]; fluorescamine for the derivatization of gentamicin, and tobramycin [40,80]; 1-naphthyl isothiocyanate for the derivatization of amikacin, and tobramycin [65,67]; fluorescein isothiocyanate for the derivatization of tobramycin [77]; o-phthalicdicarboxaldehyde for the derivatization of gentamicin [42]; 2,4,6-trinitrobenzene sulfonic acid for the derivatization of tobramycin, and amikacin [46,47]; ;-naphthoquinone-4-sulfonate for the derivatization of streptomycin [49]; ninhydrin for the derivatization of streptomycin [51]; and 9-fluorenylmethylchloroformate for the derivatization of gentamicin, neomycin, netilmicin, sisomicin, isepamicin, and amikacin [16,60,63,78,82] were other derivatizing agents used in the bioanalysis of aminoglycosides.

Challenges associated with UV and fluorescence detections (need for chromophore or fluorophore necessitating derivatization) can be circumvented using mass spectrometry. Also, mass spectrometry can analyze small sample volumes with high precision, sensitivity, and selectivity [68,81]. In this review, 15 articles reported the use of mass spectrometry as a detector for the bioanalysis of neomycin, streptomycin, dihydrostreptomycin, amikacin, kanamycin, paromomycin, tobramycin, spectinomycin, apramycin, hygromycin, gentamicin, and arbekacin [5,31,33-35,68,69,72,73,75,79,81,83-85]. Interestingly, articles published between 1977 [20] to 2002 [67] were dominated by fluorescence or UV detection methods. More importantly, mass spectrometry was the mostly used detection mode from 2014 to 2020 [34,35,81,83-85], except in one case [82]. This is not surprising as mass spectrometry appears to be an effective detection method [103].

In this review, HPLC coupled with resonance Rayleigh scattering detection was used in analyzing three aminoglycosides; amikacin, netilmicin, and etimicin [32]. An advantage of the resonance Rayleigh scattering detector over other spectroscopic techniques is that the detection limit is lower by several orders of magnitude [104].

The pulsed electrochemical detector is mostly used to analyze carbohydrates and polyalcohol. They are also used in analyzing amines, amino acids, and sulphur-containing compounds [105, 106]. One (1) article reported the use of HPLC coupled with the pulsed electrochemical detector in the bioanalysis of amikacin [15]. This approach was used to address the shortfall of using derivatization for the detection of aminoglycosides [15].

Chemiluminescence allows the detection of analytes at ultra-high sensitivity. In this review, 1 article reported the use of chemiluminescence for the detection of amikacin, and the chemiluminescence reagent used was luminol in combination with hydrogen peroxide and Cu^2+^ [71].

HPLC coupled with evaporative light scattering detector is rapidly becoming a quasi-universal detector, mitigating the need for derivatization of non-absorbing analytes. In this review, one article reported the use of an evaporative light scattering detector for the direct determination of tobramycin [70].

## Performance metrics

Performance metrics are quantifiable terms that indicate the quality of an analytical process. Some performance metrics include specificity, sensitivity, linearity, the lower limit of quantification (LLOQ), limit of detection (LOD), precision, accuracy, and calibration range. In this review, all the 71 articles reported some aspects of method performance characteristics. Method performance characteristics reported by most articles included calibration range, linearity, recovery, repeatability, and intermediate precision (Table 2). Out of the 71 articles reviewed, only four reported on matrix effect [63,83-85]. Resolution was reported by one article [75].

The calibration range is often obtained from a calibration curve [107]. Sixty-seven (67) articles reported calibration ranges, whilst 4 did not [5,23,59,79] (Table 2). These calibration ranges of HPLC varied significantly for the various drugs assayed, with some in the range of 0.1-0.5 ng/mL [14] and others 1250-200000 ng/mL [83]. Of the 20 aminoglycosides reported, only eight had established a therapeutic reference range. The reported calibration range of some of the analytes reported was outside the established therapeutic reference range [14,15,24,25,30,31,34,37,48,55,57,61,65,67,71,74,75,80-82], which invalidates the measurements obtained from the bioanalysis.

The quality of a bioanalytical method is highly dependent on the linearity of the calibration curve [108]. The linearity of the calibration curve is usually expressed as a coefficient of correlation (R^2^). The coefficient of correlation close to 1 (R^2^ ≈1) is mostly considered ideal. In the current review, 61 articles reported R^2^ values for almost all analytes under consideration, whilst 10 articles did not [23,28,33,39,45,47,50,55,56,84]. The reported coefficient of correlation was between 0.964 [75] and 1.0 [48,60,76,82]. Additionally, y-intercept, the slope of the regression of line, and residual sum of squares can also be used in evaluating linearity [109]. Out of 71 articles reviewed, 39 articles reported values for both the slope of regression and the y-intercept.

Accuracy of a bioanalytical method is normally expressed as the percent recovery by the assay of the known added amount of analyte. In this study, 63 articles reported percent recoveries that ranged mostly between 61.0 % [72] to 114.0 % [5]. Two (2) articles reported recoveries of 4.9 % [28] and 36 % [76], respectively.

LLOQ is defined as the amount of analyte in a biological matrix that can be quantitatively determined with suitable precision and accuracy [110]. Thirty-six (36) articles reported LLOQ for aminoglycosides assayed between 1 ng/mL [72] to 2340 ng/mL [79]. Forty-one (41) articles also reported LOD values in the range 0.3 ng/mL [61] to 75000 ng/ml [56]. There were instances where the same article reported both the LLOQ and LOD.

Repeatability expresses the closeness of results obtained with the same sample using the same procedure, operators, measuring system, operating conditions, and location over a short period of time. Out of 71 articles, 58 reported repeatability values ranging from 0.28 % [69] to 36 % [30]. Intermediate precision also refers to laboratory variations such as different days, instruments, and analyses. Out of the 71 reviewed articles, 56 reported intermediate precision values ranging between 0.331 % [80] to 19.76 % [52], which is within the acceptable limit of 20 % [111].

## Conclusion and outlook

Despite reported nephrotoxic and ototoxic potentials of aminoglycosides, their use in clinical settings remains relevant. The current study sought to review bioanalytical methods (specifically liquid chromatography) used in the assay of aminoglycosides in biological matrices. In all, 71 articles were reviewed, and 66 of these articles reported the use of reversed-phase liquid chromatography as a bioanalytical method [7,119].

The commonest sample treatment procedures adopted in the analysis of aminoglycosides using HPLC were protein precipitation (50 %) and solid phase extraction (39 %). Surprisingly, none of the current sample preparation methods was used by any of the articles reported in this review. It will be interesting if recent sample preparation methods such as solid-phase microextraction, micro-solid-phase extraction, dispersive micro-solid-phase extraction, magnetic solid-phase extraction, microextraction by packed sorbent, stir bar sorptive extraction, spin column extraction, liquid-phase microextraction, single-drop microextraction, hollow fiber liquid-phase microextraction, dispersive liquid-liquid microextraction, molecularly imprinted solid-phase extraction, and molecularly imprinted solid-phase micro-extraction [120-122] could be applied for bioanalysis of aminoglycosides with the potential of improving method sensitivity and selectivity.

Fluorescence (50 %), UV (24 %), and mass spectrometry (21 %) were the most adopted mode of detection in the assay of aminoglycosides, according to this review. Since mass spectrometry has been established as the detection mode of choice for bioanalysis of aminoglycoside using liquid chromatography in recent years, it is strongly recommended for use except in resource-challenged countries where fluorescence or UV detection methods can be applied after derivatization.

There is the need to establish a therapeutic reference range for all the clinically reported 20 aminoglycosides since the calibration range of analytical methods for the bioanalysis of aminoglycosides should cover such a range. It was quite surprising that some of the calibration range was outside the established therapeutic reference range. It is recommended that future liquid chromatography methods for the analysis of aminoglycosides should have calibration ranges covering established reference therapeutic ranges.

Although this review is not a systematic one, the information provided is intended to serve as a comprehensive reference for related research that may involve the assay of aminoglycosides (pharmacokinetic or drug monitoring studies).

## Figures and Tables

**Table 1.** HPLC conditions for bioanalysis of aminoglycosides.

Ref	Analyte	Internal Standard	Matrix	Sample Preparation	Stationary Phase	Mobile Phase; Elution; Flow rate (mL/min)	Derivatization agent	Detection Mode
[20]	Gentamicin	n.i.	h-serum	Ion-exchange gel chromatography (SPE)	C_18_	0.2 M Na_2_SO_4_, 0.02 M sodium pentane sulfonate, and 0.1% (v/v) acetic acid in a water/methanol (97:3, v/v); ISO; 2.0	o-phthalaldehyde	Fluorescence (excitation, 340 nm; emission, KV418 filter)
[36]	Gentamicin C1	n.i.	h-serum	SPE	C_18_	methanol/water (79:21, v/v) containing 2 g/L of tripotassium EDTA; ISO; 2.0	o-phthalaldehyde	Fluorescence (excitation, 360 nm; emission, 430 nm)
	Gentamicin C1a		d-serum					
	Gentamicin C2							
[21]	Netilmicin	n.i.	h-plasma	PPT (ACN)	C_18_ (Ambient)	acetonitrile/water (95:5, v/v); ISO; 1.0	dansyl chloride	Fluorescence (excitation, 220 nm; emission, KV470 filter)
[22]	Gentamicin	N-acetyl gentamicin C1	h-serum	SPE	C_18_	0.2 mol of Na_2_SO_4_, 0.02 mol of sodium pentane sulfonate, and 17.4 mmol of acetic acid/methanol (97:3, v/v); ISO; 2.0	o-phthalaldehyde	Fluorescence (excitation, 340 nm; emission, 418 nm)
	Amikacin	Tobramycin				0.1 mol of Na_2_SO_4_, 0.02 mol of sodium pentane sulfonate, and 17.4 mmol of acetic acid; ISO; 2.0		
	Tobramycin	Amikacin				0.1 mol of Na_2_SO_4_, 0.02 mol of sodium pentane sulfonate, and 17.4 mmol of acetic acid; ISO; 2.0		
[23]	Netilmicin	n.i.	h-serum	PPT (ACN)	C_18_	0.5 mol/L Tris buffer (pH 7.9)/trimethylamine, readjusted pH 7.9 with concentrated sulphuric acid/methanol (250:10:740, v/v/v); ISO; 2.0	o-phthalaldehyde	Fluorescence (excitation filters, 7-54/7-60; emission filters, 4-76/3-72)
	Tobramycin					0.5 mol/L Tris buffer (pH 7.9)/trimethylamine, readjusted pH 7.9 with concentrated sulphuric acid/methanol (250:10:740, v/v/v); ISO; 2.0		
	Gentamicin					Methanol/tripotassium ethylene dinitrilotetraacetate (2 g/L) (79:21, v/v); ISO; 2.0		
[37]	Tobramycin	Gentamicin C2	h-serum h-urine	PPT (ACN)	CN (Ambient)	methanol/water/acetonitrile (62:35.1:2.9, v/v/v) containing 2.5 g tripotassium ethylenediaminetetraacetic acid; ISO; 1.6	o-phthalaldehyde	Fluorescence (excitation, 340 nm; emission, 418 nm)
[38]	Gentamicin C1a Gentamicin C1+C2	n.i.	h-serum	PPT (ACN)	C_18_ (RT)	1 g/L tris(hydroxymethyl)aminomethane adjusted with hydrochloric acid to pH 7/acetonitrile (30:70, v/v); ISO; 1.5	1-fluoro-2,4-dinitrobenzene	UV (365 nm)
[39]	Gentamicin	Netilmicin	h-serum	PPT (ACN)	C_18_	acetonitrile/methylene chloride/water/methanol (80:10:8:4, v/v/v/v); ISO; 4.0	Benzene sulphonyl chloride	UV (230 nm)
[40]	Gentamicin	n.i.	r-plasma rb-urine	PPT (ACN)	Cation-exchange column	acetonitrile/phosphoric acid (5 g/L) (70:30); ISO; 2.0	Fluorescamine	Fluorescence (excitation, 275 nm; emission, 418 nm)
[41]	Amikacin	n.i.	g-plasma h-plasma	PPT (MeOH)	Silica; C_18_ (60-100 °C)	acetonitrile/water (68:32, v/v); ISO; 1.0	1-fluoro-2,4-dinitrobenzene	UV (360 nm)
[42]	Gentamicin (C1, C1a, and C2)	Netilmicin	h-serum h-urine	PPT (ACN) SPE	C_8_ (25 °C)	acetonitrile/tris(hydroxymethyl)aminomethane (1 g/L) adjusted to pH 3.0 with 1M hydrochloric acid (70:30, v/v); ISO; 1.5	o-Phthalicdicarboxaldehyde	Fluorescence (excitation, 340 nm; emission, 418 nm)
[43]	Gentamicin	n.i.	h-serum	PPT	C_18_ (50 °C)	0.1 M disodium 1,2 ethanedisulfonate and 0.005 M sodium octanesulfonate adjusted to a pH 3.5 with acetic acid/acetonitrile (85:15, v/v); ISO; 0.8	o-phthalaldehyde	Fluorescence (excitation, 365 nm; emission, 440 nm)
[44]	Gentamicin Netilmicin	Tobramycin Tobramycin	h-serum	SPE	C_18_ (22 °C)	methanol/water/ethylenediaminetetra-acetic acid pH 7.2 (80:15:5, v/v/v); ISO; 1.0	o-phthalaldehyde	Fluorescence (excitation, 340 nm; emission, 455 nm)
[45]	Amikacin	Kanamycin	h-serum	SPE	C_18_	acetonitrile/water/acetic acid (470:530:1); ISO; 2.5	1-FDNB	UV (365 nm)
[46]	Tobramycin	Sisomicin	h-serum	SPE, PPT	C_18_ (50 °C)	acetonitrile/50 mmol/L phosphate buffer adjusted to pH 3.5 with phosphoric acid (70:30, v/v); ISO; 3.0	2,4,6-trinitrobenzene sulfonic acid	UV (340 nm)
[24]	Amikacin	Neamine	h-serum	SPE	C_18_ (25 °C)	74% methanol-water	o-phthalaldehyde	Fluorescence (excitation, UG 1 filter; emission, KV 418 filter).
	Tobramycin	Gentamicin C1a				80% methanol-water 0.1 M sodium acetate, pH 7.4; GRA; 1.0		
	Netilmicin	n.i.				95% methanol-water 0.2 M sodium acetate, pH 5.0; GRA; 1.0		
	Gentamicin C1 Gentamicin C1a Gentamicin C2 Dibekacin Sisomicin	Tobramycin Tobramycin Tobramycin Tobramycin Tobramycin				80% methanol-water; GRA; 1.0 0.1 M sodium acetate, pH 7.4; GRA; 1.0		
[47]	Amikacin	Kanamycin	h-serum	PPT/SPE	C_8_ (50 °C)	acetonitrile/phosphate buffer (52:48, v/v); ISO; 2.0	TNBS	UV (340 nm)
[25]	Sisomicin Netilmicin Astromicin Micronomicin	Tobramycin Astromicin Netilmicin Sisomicin	h-serum	PPT, SPE	C8; C18 (50 °C)	25 mM sodium p-toluenesulphonate; sodium perchlorateanhgjydrous; GRA; 0.8 (C_8_); 1.5 (C_18_)	o-phthalaldehyde	Fluorescence (excitation, 360 nm; emission, 450 nm)
[48]	Tobramycin Sisomicin Netilmicin	Sisomicin Netilmicin Sisomicin	h-serum	PPT (MeOH)	C_18_	0.1 M disodium 1,2-ethanedisulfonate and 0.005 M sodium octanesulfonate in water/methanol mixture (64:36, v/v), adjusted to pH 3.5 with acetic acid; GRA; 2.0	o-phthalaldehyde	Fluorescence (excitation, 365 nm; emission, 440 nm)
[26]	Kanamycin	n.i.	h-serum	PPT (3.5 % perchloric acid)	C_18_	22 mM disodium 1, 2-ethanedisulfonate and 5 mM sodium octane sulfonate in a water/acetonitrile mixture (80:20, v /v) adjusted with acetic acid to about pH 3.5; ISO; 1.5	o-phthalaldehyde	Fluorescence (excitation, 341 nm; emission, 440 nm)
	Dibekacin					37 mM disodium 1,2-ethanesulfonate and 5 mM sodium octane sulfonate in a water/acetonitrile mixture (80:20, v/v) adjusted with acetic acid to about pH 3.5; ISO; 1.5		
[27]	Streptomycin	Dihydrostptomycin	h-serum	SPE	C_18_ (50 °C)	3.76 g of sodium l-hexanesulphonate and 9.50 g of tribasic sodium phosphate dodecahydrate dissolved in water (1 L), pH 3.0 adjusted with phosphoric acid/acetonitrile (92:8, v/v); ISO; 1.0		UV (195 nm)
[49]	Streptomycin	n.i.	h-serum	PPT (3.5 % perchloric acid)	C_18_ (65 °C)	20 mM disodium 1,2-ethanedisulfonate, 5 mM sodium octanesulfonate, and 0.4 mM NQS in a water/acetonitrile (80:20, v/v), adjusted to about pH 3.3 with acetic acid; ISO; 1.5	β—ναπητηοθυιονε—4—συλϕονατε	Fluorescence (excitation, 351 nm; emission, 420 nm)
[50]	Gentamicin C1 Gentamicin C1a Gentamicin C2	n.i.	rb-serum	SPE	C_18_ (50 °C)	solvent A: 10 mM sodium sulfate, 8 mM sodium pantanesulfonate and 20 mM acetic acid; GRA; 0.8 solvent B: 60 mM sodium sulfate, 8 mM sodium pantanesulfonate and 20 mM acetic acid; GRA; 0.8	o-phthalaldehyde	Fluorescence (excitation, 345 nm; emission, 433 nm
[51]	Streptomycin	n.i.	h-serum	PPT (3.5 % perchloric acid)	C_18_ (50-95 °C)	20 mM sodium octane sulfonate, and 5 mM ninhydrin in a water/acetonitrile (80:20, v/v), adjusted to about pH 3.3 with acetic acid; ISO; 1.5	Ninhydrin	Fluorescence (excitation, 302 nm; emission, 420 nm)
[52]	Gentamicin C1 Gentamicin C1a Gentamicin C2	n.i.	h-serum	SPE	C_18_	1 % TEA solution (adjusted to pH 6.2 ± 0.1 with phosphoric acid)/methanol (79:21, v/v); ISO; 2.0	o-phthalaldehyde	Fluorescence (excitation, 260 nm; emission, 418 nm)
[6]	Netilmicin	Gentamicin C1a	h-serum g-serum	PPT (ACN)	C_18_ (25 °C)	water/acetonitrile/acetic acid (300:700:1, v/v/v); ISO; 2.2	o-phthalaldehyde	UV (365 nm)
[53]	Sisomicin	n.i.	h-DBS	SPE	C_18_	methanol/sodium 1-heptane sulfonate (2.5g)/acetic acid/water (800:200:42:208, v/v/v/v); ISO; 0.9	o-phthalaldehyde	Fluorescence (excitation, 340 nm; emission, 450 nm)
[54]	Sisomicin	n.i.	rb-serum	SPE	C_18_	30 % ethylene glycol in 0.05 M phosphate buffer (pH 7); ISO; 0.5		Fluorescence (excitation, 340 nm; emission, 455 nm)
[14]	Neomycin	n.i.	c-milk	SPE	SHP	solvent A: Ethylenediaminetetraacetic acid, tripotassium salt (2.0 gm) dissolved in 1 L water/methanol, (300:700, v/v) solvent B: Methanol; GRA; 1.7	o-phthalaldehyde	Fluorescence (excitation, 340 nm; emission, KV 418)
[28]	Isepamicin	Dibekacin	h-serum h-urine	SPE	C_18_ (25 °C)	methanol/buffer solution containing 0.01 M sodium hexanesulphonate, 0.1 M sodium sulphate and 17 mM acetic acid (10:90, v/v); ISO; 1.1	o-phthalaldehyde	Fluorescence (excitation, 338 nm; emission, 418 nm)
[55]	Amikacin	Tobramycin	h-serum	SPE	C_18_	0.2 M sodium sulphate, 0.02 M sodium pentane sulphate and 1 ml acetic acid in 1L distilled water; ISO; 1.2	o-phthalaldehyde	Fluorescence (excitation, 340 nm; emission, 418 nm)
[56]	Amikacin	n.i.	h-serum h-urine	Ultrafiltration	C_18_ (58 °C)	acetonitrile/2-methoxyethanol/tetrahydrofuran-glacial acetic acid/tris(hydroxymethyl)-amino ethane (1% aqueous solution) (41:4.52:4.24:0.21:50, v/v); GRA; Programmed flow rate	FDNB	UV (340 nm)
[57]	Amikacin	n.i.	d-plasma	PPT (10 %w/v TCA)	C_18_ (45 °C)	0.05 M Na_2_SO_4_ and 0.005 M sodium octylsulfate, pH 3.5 adjusted with glacial acetic acid/methanol (70:30, v/v); ISO; 1.5	o-phthalaldehyde	Fluorescence (excitation, 340 nm; emission, 415 nm)
[58]	Gentamicin	Netilmicin	c-milk	SPE	C_18_	1-heptanesulfonic acid (5 g) dissolved in acetic acid/water/Me0H (50:250:700); ISO; 0.5	o-phthalaldehyde	UV (330 nm)
[59]	Netilmicin	Gentamicin	h-serum	LLE	C_18_	solvent A: water/acetic acid/heptanesulphonic acid (0.1 M) (80:10:10, v/v/v) solvent B: acetonitrile; GRA; 2.0	o-phthalaldehyde	Fluorescence (excitation, 337 nm; emission, 437 nm)
[60]	Gentamicin C1 Gentamicin C1a Gentamicin C2 Gentamicin C2a	n.i.	h-serum	SPE	C_18_	acetonitrile/water (90:10, v/v); ISO; 1.0	FMOC-C1	Fluorescence (excitation, 260 nm; emission, 315 nm)
[29]	Geneticin	n.i.	m-plasma	PPT (MeOH/TCA)	C_18_ (25 °C)	solvent A: acetonitrile and water (50:50) solvent B: Acetonitrile; GRA; 1.0	DNFB	UV (340 nm)
[61]	Gentamicin	n.i.	c-milk	SPE, PPT (30 % TCA)	C_18_ (25 °C)	0.011 M pentane sulfonic acid sodium salt, 0.0056 M sodium sulphate and 0.1 % acetic acid in water/methanol (82:18); ISO; 1.5	o-phthalaldehyde	Fluorescence (excitation, 340 nm; emission, 430 nm)
[62]	Isepamicin	Dibekacin	h-serum h-urine	PPT (methylene chloride), SPE	C_18_	solvent A: 0.01 M hexanesulphonate/0.017 M (0.1 %) acetic acid in water solvent B: 0.01 M hexanesulphonate/0.0 17 M./acetic acid/0.10 M sodium sulphate/3.53 M (15%) methanol; GRA; 1.1	o-phthalaldehyde	Fluorescence (excitation, 338 nm; emission, 417 nm)
[63]	Neomycin Netilmicin Sisomicin	n.i.	h-serum	SPE	C_18_ (20-25 °C)	acetonitrile–water (90:10, v/v); ISO; 1.0	FMOC-C1	Fluorescence (excitation, 260 nm; emission, 315 nm)
[30]	Streptomycin Dihydrostreptomycin	n.i.	c-milk	SPE	C_8_ (25 °C)	0.8 g of 1,2-naphthoquinone-4-sulfonic acid (NQS) in 0.01M sodium hexane-1-sulfonic acid/Acetonitrile (880:120, v/v); ISO; 0.5	n.i.	Fluorescence (excitation, 263 nm; emission, 435 nm)
[16]	Gentamicin	Neomycin	h-serum	LLE	C18 (30 °C)	acetonitrile/water (84.5:15.5, v/v); ISO; 2.5	FMOC-C1	Fluorescence (excitation, 260 nm; emission, 315 nm)
[64]	Gentamicin C1 Gentamicin C1a Gentamicin C 2	n.i.	h-serum d-serum h-urine d-urine	SPE	C_18_ (25 °C)	acetonitrile/Tris buffer (8.3 mmol/L, titrated to pH 7.0 with HCl) (680:320, v/v); ISO; 1.2	FNDB	UV (365 nm)
[65]	Amikacin	Naphthalen	h-serum	LLE	C_8_ (70 °C)	water/acetonitrile (57:43, v/v); ISO; 0.8	NITC	UV (230 nm)
[66]	Gentamicin	Netilmicin	h-urine	SPE	C_18_	methanol/glacial acetic acid/water (800:20:180, v/v/v) containing 0.02 M sodium heptane sulfonic acid, pH 3.4; ISO; 1.0	o-phthalaldehyde	Fluorescence (excitation, 340 nm; emission, 418 nm)
[67]	Tobramycin	Anthracene	h-serum	PPT	C_18_	water/acetonitrile (50:50, v/v); ISO; 1.3	NITC	UV (230 nm)
[68]	Tobramycin	Sisomicin	h-serum	PPT (ACN)	C_18_ (25 °C)	solvent A: water containing 2 mM ammonium acetate, 0.1 % (v/v) formic acid solvent B: Methanol containing 2 mM ammonium acetate, 0.1% (v/v) formic acid) solvent C: Solvent A containing Heptafluorobutyric acid (HBFA) (10 mM)/20% Solvent B; GRA; 2.5		MS/ESI (+)
[31]	Amikacin Gentamicin Kanamycin Neomycin Paromomycin Tobramycin	n.i.	h-serum	SPE	HILIC	solvent A: acetonitrile, 2 mM ammonium acetate and formic acid (5/95/0.2, v/v/v) solvent B: acetonitrile, 2 mM ammonium acetate and formic (95/5/0.2, v/v/v); GRA; 0.6		MS/ESI (+)
[69]	Neomycin	n.i.	h-serum	SPE	HILIC	solvent A: acetonitrile/10 mM ammonium acetate/formic acid (5:95:0.2, v/v/v) solvent B: acetonitrile/10 mM ammonium acetate/formic acid (95:5:0.2, v/v/v); GRA; 0.6		MS/ESI (+)
[70]	Tobramycin	n.i.	h-serum h-urine	SPE	C_18_ (45 °C)	water/acetonitrile 55:45 containing 1.50 ml/L HFBA (11.6 mM); ISO; 1.0		ELSD
[71]	Amikacin	n.i.	h-serum h-urine	PPT	C_18_	10–2 mol/l potassium hydrogen phthalate at pH 3.35, adjusted with diluted sodium hydroxide/acetonitrile (90:10, v/v); ISO; 1.0		CL
**[72]**	Dihydrostrepto-mycin	Streptomycin	c-milk	LLE, PPT (TCA), SPE	C_18_ (30 °C)	solvent A: 20 m solvent: B 20 mM PFPA in water/acetonitrile (50/50, v/v); ISO; 0.3M PFPA in water		MS/ESI (+)
[73]	(Neomycin) Neo 1804 A7 Neo 1804 A8 Neo 1804 A9 Neo 1804 B4	Kanamycin	h-serum rb-serum			solvent A: 20 mM formic acid and 10 mM NFPA in water solvent B: 20 mM formic acid and 10 mM NFPA in methanol; GRA; 0.5		MS/ESI (+)
[74]	Amikacin	n.i.	h-serum	PPT (ACN)	C_18_	0.01M sodium acetate pH 3/acetonitrile (85:15, v/v); ISO; 1.2	7-fluoro-4-nitrobenz-2-oxa-1,3-diazole	Fluorescence (excitation, 465 nm; emission, 531 nm)
[75]	Tobramycin	Sisomicin	h-serum	PPT (ACN)	C_18_ (60 °C)	2 mM ammonium acetate (pH 3.2)/ 5% acetonitrile (95:5, v/v); ISO; 0.5		MS/ESI (+)
[15]*	Amikacin	n.i.	h-CSF	PPT (ACN/phosphate buffer)	C_18_ (40 °C)	1.5 g sodium-1-octanesulphonate, 20 g anhydrous sodium sulphate, 15 ml tetrahydrofuran, 250 ml 0.2 M phosphate buffer pH 3, water up to 1000 mL; ISO; 1.0		PED
[76]	Isepamicin	Amikacin	h-serum	PPT (ACN)	C_18_ (40 °C)	20 mM KH_2_PO_4_ containing 8 mM TEA (pH 7.0)/acetonitrile (78:22, v/v); ISO; 1.0	6-aminoquinolyl-N-hydroxy succinimidyl carbamate	Fluorescence (excitation, 250 nm; emission, 395 nm)
[77]	Tobramycin	Neomycin	h-urine	SPE	C_18_ (Ambient)	acetonitrile/methanol/glacial acetic acid/water (420:60:5:515, v/v/v/v); ISO; 1.0	FITC	Fluorescent (excitation, 490 nm; emission, 518 nm)
[5]*	Neomycin Streptomycin Dihydrostreptomycin Amikacin Kanamycin Paromomycin Tobramycin Spectinomycin Apramycin Hygromycin Gentamicin(C1) Gentamicin(C1a) Gentamicin (C20	n.i.	c-milk	SPE	C_18_ (30 °C)	phase A: acetonitrile/water (50:950, v/v) containing 20 mM HFBA phase B: acetonitrile/water (500:500, v/v) containing 20 mM HFBA; GRA; 0.3		MS/ESI (+)
**[78]**	Isepamicin	Amikacin	r-plasma	PPT (ACN)	C_8_ (30 °C)	water/acetonitrile (32:68, v/v); ISO; 1.0	FMOC-C1	Fluorescence (excitation, 265 nm; emission, 315 nm)
**[79]**	Amikacin Gentamicin	Quinoxaline	h-serum	PPT (ACN)	C_18_ (35 °C)	buffer A (water/formic acid 0.05 %)		MS/ESI (+)
**[32]**	Amikacin Netilmicin Etimicin	n.i.	h-serum h-urine	PPT (ACN)	C_18_ (30 °C)	0.1 % trifluoroacetic acid (pH 2.2); ISO; 0.4		RRSD (excitation/emission, 370 nm)
**[33]**	Arbekacin	Dibekacin	h-serum	PPT (0.3 M perchloric acid)	C_18_	water/acetonitrile each containing 0.005 %(v/v) trifluoroacetic acid and 0.1 % (v/v) formic acid; GRA; 0.7		MS/ESI (+)
**[80]**	Tobramycin	n.i.	h-plasma	PPT (ACN)	C_18_	methanol/water (60:40, v/v); ISO; 1.0	Fluorescamine	Fluorescence (excitation, 390 nm; emission, 480 nm)
**[81]**	Amikacin Gentamicin C1 Gentamicin C1a Gentamicin C12	Kanamycin B	h-plasma	PPT (TCA)	C18 (30 °C)	solvent A: purified water solvent B: acetonitrile 100 % solvent C: perfluoropentanoic acid (200 mM)/ammonium acetate (130 mM) in purified water; GRA; 0.4		MS/ESI (+)
**[34]**	Spectinomycin Tobramycin Gentamicin Kanamycin Hygromycin Apramycin Streptomycin Dihydrostreptomycin Amikacin Neomycin	n.i.	b-milk	PPT (TCA)	C_18_	solvent A: 10 mM nonfluoropentanoic acid (NFPA) solvent B: acetonitrile in 10 mM NFPA; GRA; 0.3		MS/ESI (+)
**[82]**	Amikacin	n.i.	h-serum	PPT (ACN)	C_18_ (30 °C)	acetonitrile/water (70:30, v/v); ISO; 0.4	FMOC-C1	Fluorescence (excitation, 265 nm; emission, 315 nm)
**[83]**	Neomycin	Tobramycin	g-perilymph/CSF	SPE	C_18_ (40 °C)	solvent A: 0.2 % (v/v) HFBA in water solvent B: 0.2% (v/v) HFBA in ACN; GRA; 0.3		MS/ESI (+)
**[84]**	Amikacin	Kanamycin	h-serum	PPT (0.1 % FA)	C_18_ (40 °C)	solvent A: water containing 0.1 % formic acid, 0.01 % HFBA solvent B: acetonitrile containing 0.1% formic acid and 0.01% HFBA; GRA; 0.4		MS/ESI (+)
**[85]**	Amikacin	Kanamycin	h-plasma	PPT (0.1 % FA)	C_18_ (40 °C)	solvent: purified water with 0.1 % formic acid and 0.01 % of HFBA solvent B: d acetonitrile with 0.1 0.1% formic acid and 0.01 % of HFBA; GRA; 0.4		MS/ESI (+)
**[35]**	Paromomycin	Deuterated Paromomycin acetic acid	h-plasma	PPT (20 %w/v TCA)	C_18_ (40 °C)	5 mM HFBA in water/acetonitrile (7:3, v/v) mixture (3.5 mM HFBA in the mixture); ISO; 0.4		MS-ESI (+)

h-serum, human serum; d-serum, dog serum; h-plasma, human plasma; h-urine, human urine; r-plasma, rat plasma; rb-urine, rabbit urine; g-plasma, guinea pig plasma; rb-serum, rabbit serum; h-DBS, human dried blood spot; c-milk, cow milk; m-plasma, mouse plasma; h-CSF, human cerebral spinal fluid; MeOH, methanol; CN, cyano; ACN, FA, formic acid; acetonitrile; TCA, trichloroacetic acid; RT, room temperature; ISO, isocratic; GRA, gradient; SPE, solid phase extraction; PPT, protein precipitation; LLE, liquid-liquid extraction; CL, chemiluminescence detection; EDTA, ethylene diamine tetra acetic acid; ELSD, evaporative light scattering detector; ESI: electrospray ionization; HFBA: heptafluorobutyric acid; i.d., internal diameter; MS, mass spectrometry; ESI, electrospray ionization; Na2SO4, sodium sulphate; NFPA, nonafluoropentanoic acid; n.i.: not indicated; PED: pulse electrochemical detection; UHPLC, ultra-high performance liquid chromatography; UV-VIS: ultraviolet-visible light; FDNB, 1-fluoro-2,4-dinitrobenzene; FMOC-C1, 9-fluorenylmethylchloroformate; NITC, 1-naphthyl isothiocyanate; HILIC, hydrophilic interaction liquid chromatography; RRSD, resonance Rayleigh scattering detection

*, methods that can be optimised for the analysis of all the aminoglycosides under review.

**Table 2.** Performance metrics of HPLC method for bioanalysis of aminoglycosides.

Ref.	Analyte	Matrix effect	Resolution	Range (ng/mL)	Reference range (ng/mL)	R^2^	Slope	y-Intercept	Recovery (%)	LOD (ng/mL)	LLOQ (ng/mL)	Repeatability (%)	Intermediate precision (%)
[20]	Gentamicin	n.i.	n.i.	1000-10000	5000-10000^b)^	0.997	4.72	1.36	>95	n.i.	n.i.	n.i.	n.i.
[36]	Gentamicin C1	n.i.	n.i.	0-20000	5000-10000^b^	0.99	0.87	0.62	80-105	n.i.	n.i.	4.2-5.6	6
	Gentamicin C1a				5000-10000^b^							4.4-7.5	6
	Gentamicin C2				5000-10000^b^							3.9-5.1	6
[21]	Netilmicin	n.i.	n.i.	500-10000	10000-16000^c)^	0.999	0.563	-0.004	n.i.	500	n.i.	n.i.	n.i.
[22]	Gentamicin	n.i.	n.i.	1000-10000	5000-10000^b)^	0.997	4.72	1.36	>95	n.i.	n.i.	n.i.	3.6
	Amikacin			2000-32000	15000-25000^b)^	0.9992	0.136	0.044	93			3.2	2.8
	Tobramycin			2000-15000	5000-10000^b^	0.9997	0.278	-0.024	93			2.3	3.4
[23]	Netilmicin	n.i.	n.i.	n.i.	10000-16000^c)^	n.i.	n.i.	n.i.	96.7-110	n.i.	500	<8	<8
	Tobramycin				5000-10000^b^				94.1-98.3		1000	<8	<8
	Gentamicin				5000-10000^b^				91.5-91.8		500	<8	<8
[37]	Tobramycin	n.i.	n.i.	1000-7500 (Serum)^a)^	5000-10000^b^	0.9990 (Serum)	0.1140 (Serum)	-0.00926 (Serum)	n.i. (Serum)	n.i.	200 (Serum)	3.79-5.60 (Serum)	4.83-7.91 (Serum)
				1000-7500 (Urine)^a)^	5000-10000^b^	0.9990 (Urine)	0.3440 (Urine)	-0.04060 (Urine)	83.1-94.3 (Urine)		200 (Urine)	n.i. (Urine)	3.00-6.00 (Urine)
[38]	Gentamicin C1a/Gentamicin C1+C2	n.i.	n.i.	1000-16000	5000-10000^b^	n.i.	n.i.	n.i.	83.0-84.0	n.i.	n.i.	n.i.	n.i.
[39]	Gentamicin	n.i.	n.i.	0-10000	5000-10000^b^	n.i.	n.i.	n.i.	n.i.	n.i.	200	n.i.	n.i.
[40]	Gentamicin	n.i.	n.i.	0-40000 (Plasma)	5000-10000^b^	0.9977-0.9999	2.49-4.26	-0.21 – 0.73	93	1000	n.i.	3.5	<2.0
				0-71000 (Urine)		0.9963-0.9997	1.90-2.33	-0.10 – 1.60	n.i.	1000	n.i.	n.i.	n.i.
[41]	Amikacin	n.i.	n.i.	2000-64000	15000-25000^b)^	0.9996	3.21	1.92	n.i.	n.i.	n.i.	n.i.	n.i.
[42]	Gentamicin (C1, C1a, and C2)	n.i.	n.i.	500-10000 (Serum)	5000-10000^b^	0.9990 (Serum)	n.i.	n.i.	>85 (Serum)	n.i.	500 (Serum)	2.4-10.1 (Serum)	<8 (Serum)
				500-5000 (Urine)	5000-10000^b^	0.9990 (Urine)	n.i.	n.i.	>85 (Urine)		500 (Urine)	2.4-10.1 (Urine)	<8 (Urine)
[43]	Gentamicin	n.i.	n.i.	2700-16500	5000-10000^b^	0.999	0.792	-0.126	97-103	500	n.i.	<2.5	<3.2
[44]	Gentamicin	n.i.	n.i.	0-20000	5000-10000^b^	0.98	n.i.	n.i.	80-90	500	n.i.	n.i.	3.7-5.8
	Netilmicin			0-20000	10000-16000^c)^	n.i.			80-90	500			3.6-6.9
[45]	Amikacin	n.i.	n.i.	1000-64000	15000-25000^b)^	0.993	0.92	0.02	72	n.i.	n.i.	1.5-5.3	n.i.
[46]	Tobramycin	n.i.	n.i.	1000-25000	5000-10000^b^	0.95	n.i.	n.i.	94-98.6	<200	n.i.	4.0-4.9	4.6-5.1
[24]	Amikacin	n.i.	n.i.	1000-16000^a)^	15000-25000^b)^	n.i.	n.i.	n.i.	n.i.	n.i.		5.9	<6.0
	Tobramycin				5000-10000^b^	n.i.	n.i.	n.i.				n.i.	<6.0
	Netilmicin				10000-16000^c)^	n.i.	n.i.	n.i.				n.i.	<6.0
	Gentamicin C1				5000-10000^b^	0.9998	16.6	-1.6				4.9	<6.0
	Gentamicin C1a				5000-10000^b^	0.9997	9.5	-0.7				4.9	<6.0
	Gentamicin C2				5000-10000^b^	0.9998	7.2	0.3				4.9	<6.0
	Dibekacin				n.a.	n.i.	n.i.	n.i.			n.i.	n.i.	<6.0
	Sisomicin				n.a.	n.i.	n.i.	n.i.			n.i.	n.i.	<6.0
[47]	Amikacin	n.i.	n.i.	2500-50000	15000-25000^b)^	n.i.	n.i.	n.i.	92.8-98.4	n.i.	<500	3.5-6.0	2.8-3.1
[25]	Sisomicin	n.i.	n.i.	320-22800	n.a.	0.9997	0.034	0.0023	~100	n.i.	n.i.	n.i.	2.5
	Netilmicin			170-11600^a)^	10000-16000^c)^	0.9998	0.101	0.0085	~100				2.8
	Astromicin			100-6300	n.a.	0.9997	0.244	0.00775	~100				3.1
	Micronomicin			1000-3000	n.a.	0.9998	0.082	0.0117	~100				1.9
[48]	Tobramycin	n.i.	n.i.	1850-14830	5000-10000^b^	1	0.101	-0.057	97.5-102	300	n.i.	2.0-2.2	2.3-2.5
	Sisomicin			1660-13330	n.a.	0.999	0.136	0.002	n.i.	300	n.i.	n.i.	n.i.
	Netilmicin			1630-13060^a)^	10000-16000^c)^	0.993	0.1	0.003	n.i.	300	n.i.	n.i.	n.i.
[26]	Kanamycin	n.i.	n.i.	3000-50000	15000-25000^d)^	0.999	0.979	-0.006	99	n.i.	n.i.	0.8-2.9	2.0-5.0
	Dibekacin			500-10000	n.a.	0.999	0.998	-0.04	98.5			1.6-3.1	1.9-5.6
[27]	Streptomycin	n.i.	n.i.	5000-50000	10000-35000^e)^	0.9997	0.0476	-0.0258	80	2000	n.i.	<6	<6
[49]	Streptomycin	n.i.	n.i.	5000-50000	10000-35000^e)^	0.999	0.329	0.072	100	500	n.i.	2.67-3.02	3.01-3.50
[50]	Gentamicin C1	n.i.	n.i.	0-50000	5000-10000^b^	n.i.	n.i.	n.i.	103.9	500	n.i.	<4.0	<4.0
	Gentamicin C1a				5000-10000^b^				99.5				
	Gentamicin C2				5000-10000^b^				101.1				
[51]	Streptomycin	n.i.	.ni	5000-40000	10000-35000^e)^	0.999	0.136	0.086	100	100	n.i.	1.21-2.75	2.34-2.58
[52]	Gentamicin C1	n.i.	n.i.	1000-20000	5000-10000^b^	0.991	311.4	12.84	n.i.	20	n.i.	6.05-6.76	6.49-8.87
	Gentamicin C1a				5000-10000^b^	0.995	131.6	1.5		80		5.13-11.66	7.22-19.76
	Gentamicin C2				5000-10000^b^	0.996	124.7	4.8		80		6.36-7.02	10.35-14.82
[6]	Netilmicin	n.i.	n.i.	500-40000	10000-16000^c)^	0.99	0.0354	0.0013	78-81	500	n.i.	1.8-10.9	4.2-4.4
[53]	Sisomicin	n.i.	n.i.	50-5000 (DBS)	n.a.	0.9950 (DBS)	0.6000 (DBS)	-1.4000 (DBS)	91.6	<50	n.i.	7.9	n.i.
				50-5000 (Whole blood)		0.9950 (Whole blood)	0.6690 (Whole blood)	-0.0140 (Whole blood)					
[54]	Sisomicin	n.i.	n.i.	500-5000	n.a.	0.9996	16.753	-0.694	97.5	62.5	n.i.	n.i.	n.i.
[14]	Neomycin	n.i.	n.i.	100-5000^a)^	5161-10323^f)^	0.989	n.i.	n.i.	94-102	50	n.i.	n.i.	n.i.
[28]	Isepamicin	n.i.	n.i.	100-100000	n.a.	n.i.	n.i.	n.i.	4.9±0.1	n.i.	100 (plasma)	0.5-2.5 (Plasma)	2.6-5.5 (Plasma)
											50 (Urine)	1.0-6.0 (Urine)	2.9-7.5 (Urine)
[55]	Amikacin	n.i.		25-2000^a)^	15000-25000^b)^	n.i.	n.i.	n.i.	n.i.	n.i.	25	3.9-28.4	3.2-25.1
[56]	Amikacin	n.i.	n.i.	500-75000	15000-25000^b)^	n.i.	n.i.	n.i.	96.9	500-75000	500	1.9-3.3	2.8-3.8
				10000-500000	15000-25000^b)^				92.1		10000	0.1-2.1	0.1-6.6
[57]	Amikacin	n.i.	n.i.	100-2000^a)^	15000-25000^b)^	0.9999	2450209	-42404	99.5-105	25	100	1.1-4.7	2.9-5.6
[58]	Gentamicin	n.i.	n.i.	625-80000	5000-10000^b^	>0.990	n.i.	n.i.	>90	600	n.i.	n.i.	n.i.
[59]	Netilmicin	n.i.	n.i.	n.i.	10000-16000^c)^	0.994	n.i.	n.i.	n.i.	100	n.i.	9.22	14.23
[60]	Gentamicin C1	n.i.	n.i.	200-50000	5000-10000^b^	1	118.2	-2.5	96.8	<50	n.i.	6.4-8.6	6.3-7.9
	Gentamicin C1a				5000-10000^b^	1	92.9	-3.7	99			5.9-6.0	5.7-8.9
	Gentamicin C2				5000-10000^b^	0.9999	120.7	-4.7	97.8			5.8-7.0	5.0-5.5
	Gentamicin C2a				5000-10000^b^	0.9999	72.7	-3.3	93.9			4.3-5.8	6.0-8.9
[29]	Geneticin	n.i.	n.i.	78-10000	n.a.	0.999	n.i.	n.i.	n.i.	78	n.i.	0.02	0.09
[61]	Gentamicin	n.i.	n.i.	15-60^a)^	5000-10000^b^	>0.9950	n.i.	n.i.	78-88	0.3-0.4	15	n.i.	n.i.
[62]	Isepamicin	n.i.	n.i.	100-100000	n.a.	0.99	n.i.	n.i.	83	n.i.	100	1.9-15.0	7.4
[63]	Neomycin	n.i.	n.i.	100-1000	5161-10323^f^	0.9986	191.8	-0.3	106.7	n.i.	10	4.5	3.5
	Netilmicin				10000-16000^c)^	0.9987	229.5	7.7	95.2			3.5	2.4
	Sisomicin				n.a.	9965	167.1	13	99.1			3.5	2.5
[30]	Streptomycin	n.i.	n.i.	0-2000^a)^	10000-35000^e)^	0.9955	0.000095	37.8	77.6-96.1	8	12	5.1-36.8	n.i.
	Dihydrostreptomycin				n.a.	0.9905	0.00034	17.2	81.6-106	12	8	7.3-36.9	
[16]	Gentamicin	n.i.	n.i.	200-20000	5000-10000^b^	0.9975	n.i.	n.i.	101.1-105.6	14	n.i.	2.41-2.68	2.16-3.61
[64]	Gentamicin C1	n.i.	n.i.	0-50000	5000-10000^b^	0.9990 (Plasma)	340 (Plasma)	0 (Plasma)	72 (Plasma)	n.i.	70	1.1-11 (Plasma)	2.0-7.7 (Plasma)
						0.9990 (Urine)	470 (Urine)	0 (Urine)	98 (Urine)			7.8-8.1 (Urine)	12-16 (Urine)
	Gentamicin C1a			0-50000	5000-10000^b^	0.9960 (Plasma)	183 (Plasma)	0 (Plasma)	72 (Plasma)	n.i.	100	2.1-7.7	4.8.2013
						0.9980 (Urine)	245 (Urine)	0 (Urine)	98 (Urine)			6.1-10 (Urine)	8.7-12 (Urine)
	Gentamicin C 2			0-50000	5000-10000^b^	0.9980 (Plasma)	169 (Plasma)	0 (Plasma)	72 (Plasma)	n.i.	100	4.2.2010	6.3.2012
						0.9980 (Urine)	235 (Urine)	0 (Urine)	98 (Urine)			3.1-4.2 (Urine)	14-16 (Urine)
[65]	Amikacin	n.i.	n.i.	4000-20800^a)^	15000-25000^b)^	0.998	0.0180 ± 0.0002	-0.0498 ± 0.0055	>91	400		<5.8	2.37
[66]	Gentamicin	n.i.	n.i.	500-10000	5000-10000^b^	0.998	0.4986	0.2465	94.3	75	250	2.84-5.44	4.25-6.32
[67]	Tobramycin	n.i.	n.i.	930-9340^a)^	5000-10000^b^	0.9999	0.1027 ± 0.0011	-0.0215 ± 0.0040	>99	230	n.i.	<2.1	<5.2
[68]	Tobramycin	n.i.	n.i.	0-50000	5000-10000^b^	0.9986	0.21	0.005	93-105	100	150	2.7-5.8	4.0-6.0
[31]	Amikacin	n.i.	n.i.	100-5000^a)^	15000-25000^b)^	>0.9930	n.i.	n.i.	100	n.i.	100	0.5-9.4	n.i.
	Gentamicin				5000-10000^b^				94.4-102.3			6.1-11.4	
	Kanamycin				15000-25000^d)^				92.4-104.8			0.5-12.7	
	Neomycin				5161-10323^f^				94.5-101.7			0.3-11.6	
	Paromomycin				n.a.				94.5-104.1			0.3-10.2	
	Tobramycin				5000-10000^b^				96.0-101.6			0.4-9.9	
[69]	Neomycin	n.i.	n.i.	100-50000	5161-10323^f^	0.9985	n.i.	n.i.	100	n.i.	100	0.28-7.94	6.6-7.6
[70]	Tobramycin	n.i.	n.i.	1000-38000	5000-10000^b^	>0.9992 (Plasma)	n.i.	n.i.	85.5 (Plasma)	300	n.i.	1	1.1
						>0.992 (Urine)			90.9 (Urine)				
[71]	Amikacin	n.i.	n.i.	150-20000^a)^	15000-25000^b)^	≤0.9977	n.i.	n.i.	>92	50	2000	<9	<9
[72]	Dihydrostreptomycin	n.i.	n.i.	0-200	n.a.	0.9995	0.003101	0.08303	61	0.6	1	3.7-16.0	6.0-15.7
[73]	Neomycin	n.i.	n.i.	200-50000	5161-10323^f^				66.6	n.i.	200	n.i.	4.66-8.99
	(Neo 1804 A7)					0.9964	0.0376	0.000732					
	(Neo 1804 A8)					0.9961	0.0385	0.000228					
	(Neo 1804 A9)					0.9982	0.0403	0.00054					
	(Neo 1804 B4)					0.9959	0.0303	0.000649					
[74]	Amikacin	n.i.	n.i.	50-10000^a)^	15000-25000^b)^	0.9912	n.i.	n.i.	95.15	50	100	n.i.	n.i.
[75]	Tobramycin	n.i.	n.i.	50-1000^a)^	5000-10000^b^	0.9640-0.990	7.186-8.1126	-0.7774	93-105	n.i.	50	5.6-11.4	8.3-11.1
	Gentamicin		1.7	800-4000^a)^	5000-10000^b^	0.9998			100.32	260	500		
	Neomycin		1.4	500-10000^a)^	5161-10323^f^	0.9994			99.98	100	350		
[15]	Amikacin	n.i.	n.i.	60-4000^a)^	15000-25000^b)^	0.99	357	303	≤100	n.i.	60	0.9	n.i.
[76]	Isepamicin	n.i.	n.i.	500-50000	n.a.	1	0.08	-0.064	36.1± 6.1	n.i.	500	7.5-10.8	1.7-13.0
[77]	Tobramycin	n.i.	n.i.	250-20000	5000-10000^b^	0.9989	0.0677 ±0.0118	0.0012 ±0.0873	>99	70	250	>>>>>	>>>>>
[5]	Neomycin	n.i.	n.i.	n.i.	5161-10323^f^	0.9998	n.i.	n.i.	76-97	n.i.	n.i.	3-6	5-10
	Streptomycin				10000-35000^e)^	0.9994			72-113			4-8	7-8
	Dihydrostreptomycin				n.a.	0.999			81-107			5-9	6-9
	Amikacin				15000-25000^b)^	0.9916			69-97			8-11	13-17
	Kanamycin				15000-25000^d)^	0.9985			78-103			4-8	6-10
	Paronomycin				n.a.	0.9982			70-94			7-14	12-17
	Tobramycin				5000-10000^b^	0.9981			62-89			11-14	10-16
	Spectinomycin				144000-210000^h)^	0.9953			67-92			6-9	8-12
	Apramycin				n.a.	0.9906			71-98			7-13	10-14
	Hygromycin				n.a.	0.9918			78-98			9-12	9-14
	Gentamicin (C1)				5000-10000^b^	0.9995			82 107			7-9	7-10
	Gentamicin (C1a)				5000-10000^b^	0.9991			76-114			4-10	8-11
	Gentamicin (C2)				5000-10000^b^	0.9967			70-105			5-8	7-10
[78]	Isepamicin	n.i.	n.i.	625-15000	n.a.	0.9997	1.1896	-0.2815	99.20-103.17	100	450	<5.0	<5.0
[79]	Amikacin	n.i.	n.i.	n.i.	15000-25000^b)^	0.998	n.i.	n.i.	85.2	590	2340	<11.56	<12.10
	Gentamicin				5000-10000^b^	0.998			83.6	320	630		
[32]	Amikacin	n.i.	n.i.	25-8000	15000-25000^b)^	0.9998	1584.06	1.302	99.2-100.3	18	n.i.	<4.8	<3.0
	Netilmicin			30-74000	10000-16000^c)^	0.9997	2185.55	3.026	99.4-101.2	21		<4.8	<3.0
	Etimicin			50-63000	n.a.	0.9997	2145.92	1.094	99.6-102.4	55		<4.8	<3.0
[33]	Arbekacin	_	n.i.	100-45900	15000-20000^g)^	n.i.	0.973	0.00751	91.8-103.6	n.i.	100	n.i.	n.i.
[80]	Tobramycin	n.i.	n.i.	20-200^a)^	5000-10000^b^	0.999	0.0247 ± 0.003	1.0652	98.33-101.74	5.34	16.3	0.576-0.800	0.331-0.784
[81]	Amikacin	n.i.	n.i.	300-5000^a)^	15000-25000^b)^	0.9999	n.i.	n.i.	105.5	n.i.	n.i.	2.0-2.8	1.8-12.5
	Gentamicin C1			1000-100000	5000-10000^b^	0.9988			102.9			1.4-3.2	2.0-11.0
	Gentamicin C1a			1000-100000	5000-10000^b^	0.9992			100			1.6-8.5	2.6-10.8
	Gentamicin C2			1000-100000	5000-10000^b^	0.9994			105.4			1.4-4.6	2.8-9.3
[34]	Spectinomycin	n.i.	n.i.	5-500^a)^	144000-210000^h)^	<0.9990	n.i.	n.i.	88	20	50	3.2-6.9	0.9-8.3
	Tobramycin				5000-10000^b^				95	50	125	4.1-9.9	4.3-7.5
	Gentamicin				5000-10000^b^				87	15	25	6.0-6.6	1.4-11.1
	Kanamycin				15000-25000^d)^				92	50	37.5	4.0-5.0	1.2-6.7
	Hygromycin				n.a.				87	15	125	4.8-5.7	1.6-6.2
	Apramycin				n.a.				94	50	125	3.7-6.8	1.8-4.7
	Streptomycin				10000-35000^e)^				89	30	50	4.3-5.1	1.3-6.2
	Dihydrostreptomycin				n.a.				91	20	50	4.2-6.1	1.4-6.5
	Amikacin				15000-25000^b)^				91	50	125	3.8-7.6	4.3-5.8
	Neomycin				5161-10323^f^				93	50	125	2.7-6.0	4.7-8.3
[82]	Amikacin	n.i.	n.i.	500-10000^a)^	15000-25000^b)^	1	1097931	1897528	88.02-102.56	50	n.i.	2.12-5.07	2.64-5.80
[83]	Neomycin	-0.10 – 1.33	n.i.	1250-200000	5161-10323^f^	0.9909	n.i.	n.i.	98.9-113.7	625	1250	5.50-11.9	7.0-10.4
[84]	Amikacin	-1.1	n.i.	500-100000	15000-25000^b)^	n/i	n.i.	n.i.	83.1-89.7	n.i.	500	4.7.2006	3.8-5.6
[85]	Amikacin	-7.6 - -8.8	n.i.	500-100000	15000-25000^b)^	0.99	n.i.	n.i.	82.7	n.i.	500	3.6-6.6	3.8-4.9
[35]	Paromomycin	≤12	n.i.	5-1000	n.a.	0.997	n.i.	n.i.	100	n.i.	5	≤4.3	≤2.3

R^2^, coefficient of correlation; LOD, limit of detection; LLOQ, lower limit of quantification; n.i., not indicated; n.a., not available

^a)^calibration range does not cover the therapeutic reference range

^b)^source, [112]

^c)^Source, [113]

^d)^source, [114]

^e)^source, [115]

^f)^source, [116]

^g)^source, [117]

^h)^source, [118].
